# Advancing Tuberculosis Treatment with Next-Generation Drugs and Smart Delivery Systems

**DOI:** 10.3390/pharmaceutics18010060

**Published:** 2026-01-01

**Authors:** Ayman Elbehiry, Eman Marzouk, Adil Abalkhail

**Affiliations:** Department of Public Health, College of Applied Medical Sciences, Qassim University, P.O. Box 6666, Buraydah 51452, Saudi Arabia; ar.elbehiry@qu.edu.sa (A.E.); e.marzouk@qu.edu.sa (E.M.)

**Keywords:** tuberculosis, drug-resistant tuberculosis, host-directed therapy, nanocarriers, inhaled drug delivery, long-acting formulations, precision pharmaceutics, public health

## Abstract

Tuberculosis (TB) remains a leading infectious killer, increasingly complicated by multidrug-resistant (MDR) and extensively drug-resistant (XDR) disease; current regimens, although effective, are prolonged, toxic, and often fail to reach intracellular bacilli in heterogeneous lung lesions. This narrative review synthesizes how next-generation antimycobacterial strategies can be translated “from molecule to patient” by coupling potent therapeutics with delivery platforms tailored to the lesion microenvironment. We survey emerging small-molecule classes, including decaprenylphosphoryl-β-D-ribose 2′-epimerase (DprE1) inhibitors, mycobacterial membrane protein large 3 (MmpL3) inhibitors, and respiratory chain blockers, alongside optimized uses of established agents and host-directed therapies (HDTs). These are mapped to inhalable and nanocarrier systems that improve intralesional exposure, macrophage uptake, and targeted release while reducing systemic toxicity. Particular emphasis is placed on pulmonary dry powder inhalers (DPIs) and aerosols for direct lung targeting, stimuli-responsive carriers that trigger release through pH, redox, or enzymatic cues, and long-acting depots or implants that shift daily dosing to monthly or quarterly schedules to enhance adherence, safety, and access. We also outline translational enablers, including model-informed pharmacokinetic/pharmacodynamic (PK/PD) integration, device formulation co-design, manufacturability, regulatory quality frameworks, and patient-centered implementation. Overall, aligning stronger drugs with smart delivery platforms offers a practical pathway to shorter, safer, and more easily completed TB therapy, improving both individual outcomes and public health impact.

## 1. Introduction

Tuberculosis (TB) remains a major global health threat. In 2023, an estimated 10.8 million people (95% uncertainty interval, 10.1–11.7 million) developed TB and 1.25 million died, keeping TB among the leading infectious causes of death worldwide [1]. Independent analyses from the Global Burden of Disease program show that declines in incidence are too slow to offset population growth, with the highest burden in sub-Saharan Africa and South-East Asia [2]. Structural factors, including socioeconomic inequity and delayed diagnosis, continue to drive transmission and mortality. These constraints highlight the need to strengthen health systems and improve access to timely, high-quality care [3]. They also point to a practical conclusion: next-generation antimycobacterial agents must be paired with smart delivery platforms that raise drug exposure at sites of bacillary persistence while reducing systemic toxicity so treatment remains effective and finishable for patients [1].

Drug-resistant tuberculosis (DR-TB) remains a major global challenge. In 2023, multidrug-resistant or rifampicin-resistant TB accounted for 3.2% of new cases and 16% of previously treated cases worldwide [1]. Despite this burden, access to effective treatment remains limited. The World Health Organization (WHO) estimated that about 400,000 people developed MDR/RR-TB globally in 2023, but only around 176,000 were diagnosed and started on appropriate therapy [1,4]. This gap reflects ongoing limitations in drug-susceptibility testing, health system capacity, and access to care. Modeling studies indicate that without faster progress in prevention, diagnosis, and treatment, TB, including drug-resistant forms, will continue to cause avoidable illness and death [2]. These trends reinforce the need for improved drugs and delivery approaches that can reach more patients and support treatment completion. Scalable long-acting and pulmonary delivery systems promoted by the WHO may help reduce these gaps by simplifying treatment and supporting decentralized care [1].

For drug-susceptible TB, the WHO-recommended six-month multidrug regimen is highly effective but remains burdensome due to toxicity, pill load, and daily adherence requirements [5,6]. Shorter options exist, including the four-month rifapentine and moxifloxacin regimen, which was noninferior to the six-month standard in Dorman et al. [7]. Yet real-world completion can be limited by adverse effects, drug–drug interactions, and implementation challenges. Analyses by Carr et al. [8] and guidance from the National Tuberculosis Controllers Association highlight constraints such as rifapentine cost, interaction risks with antiretroviral therapy, and the need for strong toxicity-monitoring systems. A central biological barrier is insufficient drug exposure at sites of bacillary persistence. Lesion-based studies show heterogeneous and often low penetration of key agents into intracellular compartments and caseating granulomas, conditions that favor relapse and persistence [9,10,11,12]. These clinical and biological findings support delivery strategies that improve intralesional and intracellular drug levels while reducing systemic toxicity. Pregnant individuals with human immunodeficiency virus (HIV)/TB coinfection face increased maternal, fetal, and infant risk, further emphasizing the value of delivery systems that simplify dosing, reduce drug–drug interactions, and improve completion [13].

Pulmonary TB forms a range of lesions, including cellular granulomas with caseous, hypoxic, protein-rich necrotic centers that hinder diffusion and lower effective drug concentrations where *Mycobacterium tuberculosis* (*M. tuberculosis*) persists [11]. Caseous caseum forms acidic, lipid-rich microenvironments that promote bacterial tolerance and restrict antibiotic activity [14]. High-resolution mass-spectrometry imaging and rabbit histopathology show that fluoroquinolone penetration varies with necrosis, distance from the lesion edge, and macrophage density [12]. Human resection studies measuring drug levels across nine pulmonary lesion types confirm underexposure to several first-line agents and propose links between lesion penetration and resistance emergence [15]. These lesion-centered insights, supported by lesion-penetration modeling from Sarathy et al. [9], provide a rationale for pulmonary and macrophage-targeted delivery strategies that ensure effective exposure in necrotic and cavitary compartments.

Historically, streptomycin demonstrated that TB could be cured but also revealed the limitations of monotherapy, as resistance emerged early in clinical use [16]. These findings led to the early adoption of combination therapy, including the addition of para-aminosalicylic acid to reduce resistance [17]. The subsequent introduction of isoniazid, pyrazinamide, ethambutol, and rifampicin enabled the development of standardized multidrug chemotherapy regimens that form the backbone of modern TB treatment [18,19]. Rifampicin, discovered in the mid-1960s and approved in 1968, provided potent sterilizing activity that transformed combination therapy and shortened treatment duration [20,21].

After decades without new drug classes, bedaquiline (BDQ) received U.S. Food and Drug Administration accelerated approval in 2012, enabling all-oral regimens for DR-TB [22]. Delamanid (DLM) obtained European Union marketing authorization in 2014 [23]. In 2019, pretomanid (Pa) was approved as part of the BPaL regimen, providing a shorter, all-oral option for selected patients with highly drug-resistant disease [24]. This evolution illustrates a central theme in tuberculosis care: sustained progress depends on combining potent drugs with strategies that support effective delivery, adherence, and patient convenience.

Even strong agents face barriers related to biopharmaceutics, host factors, and lesion heterogeneity, which limit the fraction of free, active drug that reaches intracellular bacilli in granulomas [11]. Inhaled administration can raise pulmonary concentrations without proportionally raising systemic toxicity, as shown in recent reviews of inhaled TB therapy [25,26]. Nanocarriers, including liposomes, solid-lipid nanoparticles (LNPs), polymeric systems, and hybrids, can stabilize labile drugs, prolong residence, and target alveolar macrophages or granulomatous tissue [27,28]. Stimuli-responsive platforms triggered by pH, enzymes, or redox gradients allow site-specific release in diseased lungs [25]. Advances by Aekwattanaphol et al. [29] and Patil et al. [30] demonstrate inhalable Pa and BDQ-Pa nanoparticle dry-powder formulations developed through quality-by-design principles. Macrophage-targeted platforms, such as mannose-decorated solid-LNPs [31] and glucan-based carriers [32], significantly enhance drug accumulation in alveolar phagocytes while maintaining safety.

Despite substantial progress, major gaps remain in human evidence. Host-directed therapies (HDTs) show encouraging signals but lack definitive randomized data and clear dosing windows. Many nano- or inhaled platforms that improve exposure in animals have not yet demonstrated faster culture conversion or lower relapse compared with optimized oral therapy in humans. Until such outcomes are shown, the idea that inhaled or nanoformulations are superior should remain a testable hypothesis rather than an established conclusion [25,33].

Preclinical data support both dry-powder inhalers and nebulized formulations for direct lung delivery, including new inhaled Pa candidates [25]. Macrophage-directed solid-lipid systems concentrate drug at bacillary niches in the deep lung and raise local exposure [27]. Long-acting depots and implants are also being explored to sustain therapeutic levels and support adherence, with translational models described for both preventive and therapeutic settings [34]. Advances in computational fluid dynamics and imaging now enable prediction of aerosol deposition and intrapulmonary distribution, supporting rational device formulation co-design [35,36]. Recent outputs from the Long-Acting/Extended-Release Antiretroviral Resource Program [37] define target-product profiles, regulatory considerations, and community preferences for long-acting TB therapy. Vermeulen et al. [38] show strong patient and provider support for monthly or quarterly injectable formulations, suggesting that practical long-acting products could improve adherence and public-health outcomes. LNP messenger RNA (mRNA) platforms are also emerging for TB. Early findings show promising immunogenicity, but efficacy, durability, and scalable manufacturing for TB-specific use remain to be demonstrated [39].

**Figure 1:**
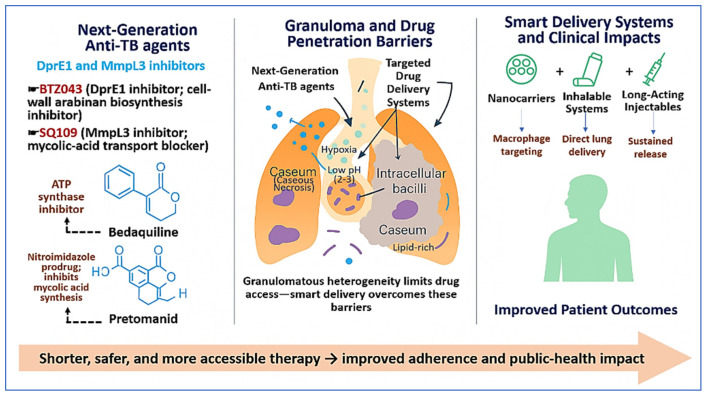
Routes of administration, lesion-associated PK barriers, and delivery strategies for next-generation anti-TB therapies. Next-generation agents, including BTZ043 (DprE1 inhibition), SQ109 (MmpL3 inhibition), bedaquiline (ATP synthase inhibition), and pretomanid (nitroimidazole prodrug), target essential pathways in *M. tuberculosis*. Following administration, drug distribution is strongly influenced by pulmonary lesion architecture. Granulomas commonly contain caseous necrosis, lipid-rich regions, hypoxia, and low pH, all of which limit drug penetration and reduce exposure at intracellular and intralesional bacterial sites. The schematic illustrates how delivery approaches such as nanocarriers, inhalable formulations, and long-acting injectables are designed to overcome these PK barriers by improving lung targeting, macrophage uptake, and sustained local drug release. These strategies aim to enhance therapeutic efficacy while reducing systemic toxicity and supporting shorter, safer, and more accessible treatment regimens.

Across these developments, progress depends on linking novel molecular entities with innovative delivery systems that balance intralesional exposure with toxicity [40]. Targets such as decaprenylphosphoryl-β-D-ribose 2′-epimerase (DprE1), essential for arabinan biosynthesis in the mycobacterial cell wall [41], and mycobacterial membrane protein large 3 (MmpL3), a lipid transporter that supports mycolic-acid trafficking and cell-wall integrity [42], represent opportunities that modern chemistry and formulation science can advance. This narrative review integrates next-generation therapeutics with advanced delivery strategies to show how pharmaceutical innovation may shorten therapy, reduce toxicity, improve adherence, and expand access. Pulmonary and nanocarrier approaches, stimuli-responsive and sustained-release technologies, and translational dimensions including scalable manufacturing, quality and regulatory pathways, and equitable access will shape whether scientific progress reaches the people who need it. Figure 1 provides a visual overview of the progression from drug administration to lesion-level exposure in TB therapy. It integrates next-generation anti-tuberculosis drug mechanisms with pulmonary lesion biology to illustrate how pharmacokinetic (PK) barriers within granulomas limit effective drug delivery. The figure highlights how advanced delivery strategies, including inhalable systems, nanocarriers, and long-acting formulations, are intended to improve intralesional and intracellular exposure. This framework supports the central premise of the review: that aligning drug design, delivery strategy, and lesion-site PKs is critical for improving treatment outcomes.

## 2. Materials and Methods: Literature Search and Review Approach

This article is a narrative review that examines recent advances in TB drug therapy and delivery systems. Vaccines and infection-control measures are outside the scope of this review. The focus is on pharmaceutical strategies that support TB treatment from drug discovery to patient care. The literature search was carried out using PubMed, EMBASE, and ClinicalTrials.gov. We also reviewed selected documents from regulatory agencies, including the U.S. Food and Drug Administration and the European Medicines Agency. Publications from 2006 to October 2025 were considered.

Search terms combined TB with nanocarrier, inhaled delivery, long-acting formulations, DprE1, MmpL3, host-directed therapy, and macrophage. We also examined reference lists of relevant articles to identify additional studies. Priority was given to human clinical studies, translational pharmacokinetic and pharmacodynamic research, and regulatory or manufacturing-related sources. Preclinical studies were included when they helped explain how drugs reach TB lesions or infected cells.

## 3. New Horizons in Anti-Tuberculosis Drug Discovery

A new therapeutics era is emerging. Potent small molecules, refined repurposing, host-directed strategies, and early biologic or genetic approaches are being developed to reach bacilli in hypoxic, acidic, and caseous niches while keeping treatment tolerable enough to complete [43,44]. Beyond BDQ, DLM, and Pa, the clinical pipeline includes DprE1 inhibitors such as quabodepistat (OPC-167832), benzothiazinones including BTZ043 and macozinone, and TBA-7371. Other candidates include respiratory-chain blockers such as telacebec (Q203) and protein-synthesis inhibitors such as the leucyl-tRNA synthetase inhibitor ganfeborole. Real-time genomics is clarifying resistance patterns and improving regimen design [45,46,47]. The translational goal is to pair strong mechanisms with delivery that reaches the lesion microenvironment without adding undue toxicity so cures remain durable and resistance slows [48].

Recent progress in TB drug development has produced a diverse group of new and emerging agents that act on distinct and essential mycobacterial pathways. These drugs differ not only in their molecular targets but also in their stage of clinical development and current therapeutic role. For clarity, Table 1 provides a concise overview of the most important next-generation anti-tuberculosis drugs, summarizing their drug class, primary mechanism of action, and current clinical status or approved use. This overview is intended to support the detailed discussion that follows by allowing readers to quickly orient themselves within the evolving therapeutic landscape before individual compounds and strategies are examined in depth.

### 3.1. Innovative Small Molecules

BDQ remains a cornerstone, but resistance mediated by *Rv0678* or *mmpR5* variants is increasingly recognized. These variants often show cross-resistance with clofazimine (CFZ), and *atpE* mutations appear less frequently. Rapid molecular drug-susceptibility testing and careful companion selection are essential [50,51,52]. DLM and Pa require F420-dependent activation through *ddn*, *fgd1*, *fbiA*, *fbiB*, and *fbiC*. Mutations along this pathway can inactivate the class and undermine salvage regimens [55,56]. Reports of high-level Pa resistance linked to specific *ddn* variants support baseline and on-treatment genotyping where feasible [57]. These findings show that strong drugs have fragile points. *Rv0678* upregulates the MmpL5 efflux pump and drives BDQ-CFZ cross-resistance. Defects in the F420 pathway, including *ddn*, *fbiA*, *fbiB*, *fbiC*, and *fgd1*, threaten DLM and Pa. Until more human data across these resistance backgrounds become available, rifamycins remain dependable anchors [55,65].

Nitrofuran derivatives are gaining renewed attention in anti-TB research. Recent medicinal chemistry studies have focused on 5-nitrofuran-2-yl scaffolds that show measurable activity against *M. tuberculosis* and may serve as starting points for further optimization [66]. Modified nitrofurantoin analogs with improved lipophilicity and reduced protein binding have demonstrated stronger antimycobacterial activity in vitro than the parent drug [67]. In addition, more complex nitrofuran-based hybrids have shown potent inhibition of both replicating and non-replicating *M. tuberculosis*, with favorable selectivity indices [68]. Other newly synthesized nitrofuran-containing compounds have also displayed activity against *M. tuberculosis*, supporting continued investigation of this class [69]. Although clinical evidence is not yet available, these results support the inclusion of nitrofuran derivatives as exploratory preclinical candidates in tuberculosis drug development.

DprE1 inhibitors continue to progress toward clinical use. A randomized 14-day study showed early bactericidal activity and supportive pharmacokinetics for quabodepistat combinations. Phase 2 evaluation of BTZ043 showed on-target bactericidal activity with a defined safety profile [46,60]. Dose-dependent early bactericidal activity for TBA-7371 and continued work with benzothiazinones strengthen DprE1 as a second pillar beyond rifamycins. Exposure-response modeling supports dose selection [46,48,70]. Work targeting MmpL3 is grounded in strong biology but has faced uneven oral exposure, including the low bioavailability and first-pass metabolism of SQ109. Q203, which inhibits the cytochrome bc1 complex through QcrB, shows dose-dependent early bactericidal activity, although its optimal combination partners are still being defined. Much of the understanding of lesion-site exposure comes from translational models and imaging rather than direct human measurements, so enthusiasm remains ahead of clinical certainty [9,60,71].

MmpL3 remains an attractive target because it mediates trehalose monomycolate export and supports cell-wall integrity. SQ109 can allosterically trap MmpL3 in a nonproductive state, revealing proton-coupled transport as a modifiable weakness with natural synergy alongside inhibitors of energy metabolism [53,72]. New chemotypes sustain interest in MmpL3 despite earlier limitations with bioavailability [42]. Q203 continues to show Phase 2a, dose-dependent early bactericidal activity and appears compatible with inhibitors of electron transport or ATP synthesis. New gyrase-directed scaffolds, including GyrB-focused agents and novel bacterial topoisomerase inhibitors, expand options against fluoroquinolone resistance [63,73,74,75].

### 3.2. Drug Repurposing and Adjunctive Antibacterials

Linezolid (LZD) remains central to short, all-oral regimens for drug-resistant TB. Studies such as Nix-TB and ZeNix show that reducing dose or duration can sustain efficacy while lowering the risk of neuropathy and myelosuppression [58,59]. However, these adverse effects remain common and clinically important. Patients receiving linezolid should therefore undergo regular clinical assessment for peripheral neuropathy and visual symptoms, along with routine blood count monitoring to detect anemia or thrombocytopenia early, as recommended in international treatment guidance and supported by clinical safety analyses [49,76]. Careful monitoring is essential to allow timely dose adjustment or interruption when toxicity develops. Next-generation oxazolidinones aim to preserve sterilizing activity with a wider safety margin. Sutezolid shows early bactericidal activity and combination promise, and TBI-223 shows Phase 1 pharmacokinetics consistent with clinically relevant exposure [62,77,78,79,80]. Phase 3 comparisons are pending, so claims of improved safety with equal efficacy remain provisional until patient-centered endpoints are aligned [77,81]. Until more definitive clinical data are available, similar safety monitoring should be applied when newer oxazolidinones are used in TB regimens [76].

CFZ improves outcomes in pooled analyses but requires monitoring for corrected QT interval prolongation and for *Rv0678*-linked cross-resistance with BDQ [82,83]. For drug-susceptible TB, the four-month rifapentine–moxifloxacin regimen (Study 31/A5349) is noninferior to the six-month standard [7]. For drug-resistant TB, Opti-Q supports levofloxacin 1000 mg per day to reach PK targets when fluoroquinolones remain active [84]. Carbapenem and beta-lactamase-inhibitor combinations, including meropenem or imipenem with clavulanate, are not first-line but can serve as salvage options. Early cohorts suggest that meropenem with clavulanate may offer an advantage with acceptable toxicity, although larger studies are needed [85,86].

### 3.3. Host-Directed Therapies (Hdts)

Across several observational datasets, metformin use is associated with lower all-cause mortality during tuberculosis treatment. This association is consistent with activation of AMP-activated protein kinase and increased mitochondrial reactive oxygen species (ROS), which promote phagosome maturation and antibacterial autophagy [87,88,89]. Statins may also enhance autophagy and phagolysosome maturation, although clinical evidence remains mixed [90,91,92]. Vitamin D is the most extensively studied immunonutrient. Randomized trials and meta-analyses show faster smear or culture conversion mainly in individuals with baseline vitamin D deficiency. In patients with sufficient levels, benefits are modest or absent, supporting a targeted rather than universal approach to supplementation [93,94].

Several host-directed approaches are advancing. Modulation of the mTOR pathway, including rapamycin, can increase antimycobacterial autophagy and improve myeloid function in models and ex vivo human macrophages. Dosing must be managed carefully to avoid immunosuppression [95,96]. Nitazoxanide affects host pathways and pathogen metabolism. It has reached early bactericidal testing and continues to be evaluated with formulation work to optimize PKs [33,97]. Amiodarone induces autophagy and reduces mycobacterial burden in macrophage systems and zebrafish, although any repurposing would require careful safety evaluation [98]. Overall, signals are encouraging, but dosing windows, drug–drug interactions, and consistent endpoints must be defined before routine use, especially among individuals with human immunodeficiency virus, diabetes, or pregnancy [33,99]. Materials science may increase precision. Macrophage-targeted nanosystems can intensify autophagy, concentrate drugs in granuloma-resident phagocytes, and act synergistically with antibiotics. These approaches may increase lesion exposure without raising systemic toxicity [32,100].

### 3.4. Emerging Biologic and Genetic Therapies

Peptide-based strategies are progressing. The human cathelicidin LL-37 kills *M. tuberculosis* and modulates macrophage responses through autophagy induction, cytokine balance, and improved differentiation. Small-molecule inducers and nanocarriers aim to protect LL-37 and guide it toward granuloma macrophages [101,102,103,104]. Antibody-based approaches are also moving forward. A human monoclonal antibody to LpqH showed protective activity in human ex vivo systems and mice with isotype-dependent effects. These findings support continued interest in antibody drug conjugates (ADCs) for TB [105].

Phage therapy is more developed for nontuberculous mycobacteria, but TB-specific findings are now emerging. The mycobacteriophage DS6A kills *M. tuberculosis* in vitro, in primary human macrophages, and in humanized mice. Enzymes such as LysB lyse drug-resistant strains. Delivery, immunity, and manufacturing remain major challenges before formal trials can begin [106,107,108,109]. CRISPR interference screens identify vulnerable nodes and potentiation targets. Cas13-based diagnostics detect TB DNA or RNA rapidly, including plasma cell-free DNA [110,111,112,113]. LNP-mRNA platforms encoding fusion TB antigens such as CysVac2 induce strong T-cell responses and alter early innate trafficking in mice. Chemistry is being adapted for safe pulmonary or systemic dosing [114,115,116]. These biologic and genetic approaches remain early in translation. Immune reactivity, scalable manufacturing, regulatory pathways, and penetration into granulomas continue to limit progress. Most efficacy signals remain preclinical. Questions about durability, repeat dosing, and added value beyond standard antibiotics in humans remain open [39,117].

Table 2 summarizes the direction of TB therapeutics. It presents goals, mechanisms, and translational progress for major agents. Established drugs appear alongside new scaffolds, refined repurposing strategies, and biologic or host-directed concepts. Each entry includes a concise rationale and a key study for reference. Collectively, these developments reflect a shift from chemistry alone toward chemistry combined with immune modulation and targeted delivery so therapy can become shorter, safer, and easier to complete.

## 4. Innovative Delivery Systems in Tb Pharmaceutics

The future of TB care depends on how well drugs reaches the bacilli. Even potent agents can underperform when they fail to penetrate macrophages, hypoxic caseum, or fibrotic granulomas. Delivery science is narrowing this gap. Inhaled systems place drug where disease is concentrated. Nanocarriers guide payloads into the cells that matter [129,130]. Stimuli-responsive materials release drug only in lesion environments. Long-acting depots reduce pill burden. Co-delivery platforms unite antibacterial and host-directed actions in a single system. These approaches aim to raise intralesional exposure, reduce systemic toxicity, and support treatment completion.

### 4.1. Pulmonary and Inhalable Delivery Systems

Direct lung targeting is a logical approach for pulmonary TB. It increases local concentrations, bypasses first-pass metabolism, and moves drug into alveolar macrophages. Across preclinical studies, inhaled nano- and micro-formulations raise lung and intracellular exposure while reducing systemic levels. Ramachandran and colleagues summarize this trend. Inhalable NPs improve efficacy and reduce adverse effects in animal models, and clinical work is beginning to advance [131].

**Figure 2:**
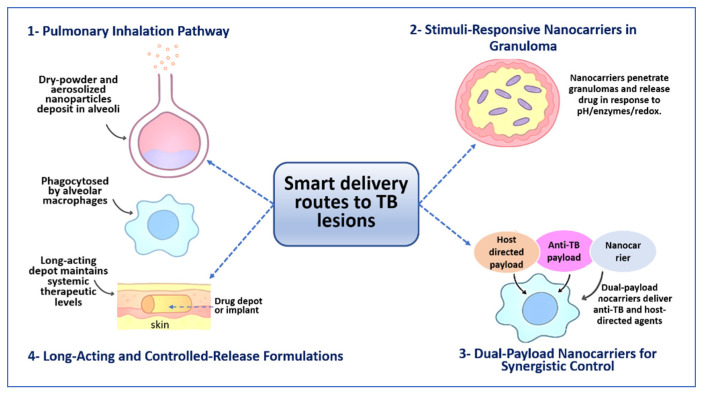
Delivery routes and formulation strategies for targeting TB lesions. The schematic illustrates how advanced drug delivery systems are designed to transport anti-TB agents to sites where *M. tuberculosis* persists. Inhaled dry powders and aerosolized NPs deposit in the alveoli and are taken up by alveolar macrophages, increasing drug exposure at the primary site of infection. Stimuli-responsive nanocarriers penetrate granulomas and release their payload in response to local conditions such as acidic pH, enzymatic activity, or redox gradients. Dual-payload systems combine antibacterial agents with host-directed compounds to enhance intracellular killing and modulate host responses. Long-acting depots or controlled-release formulations maintain therapeutic concentrations over extended periods, reducing dosing frequency and supporting sustained lesion-level exposure.

To clarify how particle composition and physicochemical characteristics influence lung deposition, cellular uptake, drug release, and therapeutic performance, Table 3 summarizes key design features of inhalable delivery systems relevant to TB treatment. Figure 2 illustrates how different routes of administration and formulation strategies are used to deliver anti-TB drugs to infected lung tissue and intracellular niches, highlighting inhalation-based delivery, lesion-responsive nanocarriers, dual-payload systems, and long-acting formulations.

Dry-powder inhalers are well suited for low-resource settings because they are portable and do not require power. Their success depends on precise engineering of particle size, moisture resistance, and the match between device and formulation. Two translational examples stand out. Aekwattanaphol et al. [29] developed a proliposomal Pa dry powder with respirable particles and a defined release profile. Patil et al. [30] used quality-by-design (QbD) methods to produce an inhalable BDQ–Pa nanoparticle powder with strong aerosol performance and additive or synergistic activity against *M. tuberculosis*.

Nebulized systems serve patients who cannot generate adequate inspiratory flow. Liposome-based aerosols are promising. Liposomal rifampicin distributes uniformly across the lung and can deliver bactericidal exposures at a fraction of oral doses in preclinical studies. Reviews describe how lipid vesicles improve solubility, residence, and tolerability in lung disease, including TB [135,136]. A broad review of inhaled anti-TB powders highlights strong animal data and early human dosing studies now underway [25].

Clinical limitations remain. Dry-powder inhalers require adequate inspiratory flow and humidity-resistant formulations. Nebulizers add time and require maintenance. In preclinical work, inhaled rifampicin often achieves higher lung exposure at lower doses, yet human trials have not demonstrated faster culture conversion or reduced relapse compared with optimized oral therapy. Thus, inhaled therapy remains a promising but unproven hypothesis [25,137].

### 4.2. Nanocarrier-Based Systems

Nanocarriers play a central role in modern TB drug delivery. Platforms such as poly(lactic-co-glycolic acid) NPs, solid LNPs, nanostructured lipid carriers, liposomes, dendrimers, and hybrid particles are used to protect drugs, control release, and improve delivery to macrophages and granulomatous lesions. Systems with sizes below 200 nm and with macrophage-recognition ligands consistently show higher cellular uptake and stronger intracellular activity than free drugs [28].

Polymeric carriers such as PLGA offer predictable degradation and enable surface functionalization with targeting ligands, including mannose and glucan. Lipid-based systems are well suited for hydrophobic agents and can improve drug stability, extend circulation time, and reduce systemic toxicity. PEGylation combined with macrophage-targeting ligands further enhances uptake in the alveolar compartment. Recent reviews emphasize both the potential of these systems and their limitations, including constraints related to drug loading, formulation stability, scale-up, and regulatory requirements. Mannose-engineered and hyaluronic acid linked particles that preferentially target infected phagocytes are well documented examples [28].

Beyond size and surface charge, deliberate surface modification is widely used to enhance macrophage targeting. Functionalization with ligands such as mannose promotes receptor-mediated uptake and improves intracellular delivery of anti-TB agents [138]. Peptide-based modifications are also under active investigation. Short targeting peptides and antimicrobial peptides can be designed to enhance cellular internalization, facilitate endosomal escape, or engage macrophage receptors, leading to increased cellular uptake and improved local bioavailability in the lung [139]. Although most peptide-modified nanocarriers remain at an early stage of development, they provide practical and adaptable strategies for macrophage-directed delivery. Studies in preclinical systems show that functionalization with receptor-specific peptides increases targeted cellular localization and internalization by phagocytic cells, supporting their potential application in TB nanocarriers [140].

Special consideration is required for pediatric populations. Child-friendly nanotechnologies administered by inhalation or oral routes may improve treatment acceptability and adherence [141]. Translation of nanocarrier systems to human use remains challenging. Small changes in particle size or surface chemistry can markedly alter biodistribution and exposure. Regulatory agencies increasingly require clear links between critical quality attributes and clinical performance. Issues related to batch comparability and cost of goods are often insufficiently addressed. In addition, because human PK and PD assessments rely largely on plasma measurements rather than lesion-level data, evidence that nanocarriers improve clinical outcomes remains indirect in the absence of validated biomarkers of intralesional drug delivery [28,142].

### 4.3. Stimuli-Responsive and Smart Delivery

TB lesions provide cues that can trigger targeted release, including acidic pH within phagosomes and caseum, redox gradients, and lesion-associated enzymes. pH-responsive designs such as chitosan-coated PLGA or acid-cleavable linkers remain inert at physiological pH and release drug in the acidic environment of infected macrophages. These strategies can increase intracellular activity while limiting off-target exposure [143,144]. Redox-responsive carriers use disulfide or thioketal bonds that respond to intracellular ROS, further biasing release toward infected cells. Some designs integrate multiple triggers, pairing pH-sensitive shells with redox-cleavable cores. Reviews describe rapid growth in responsive materials from 2023 through 2025 [145]. Table 4 summarizes the main classes of stimuli-responsive delivery systems relevant to tuberculosis lesions, highlighting the dominant biological triggers, common design features, and the rationale for their use in targeted drug release.

Biological heterogeneity remains a key challenge. Phagosomal pH and redox gradients vary across granuloma types and evolve over time. A trigger that performs well in one niche may misfire in another. Stacking triggers may improve precision but adds manufacturing and regulatory complexity. Human data comparing responsive systems with simpler sustained-release approaches are still limited [146,147]. Pulmonary targeting complements responsive release. A 2024 overview describes how inhalable nanoparticle powders can be engineered to deposit in alveoli and then release drug responsively within macrophages, increasing lesion exposure while avoiding systemic toxicity [148].

### 4.4. Long-Acting and Controlled-Release Formulations

Daily therapy for several months is difficult in all settings. Long-acting platforms aim to convert daily dosing into monthly or quarterly administration through injectable depots, implants, or controlled-release oral formulations. Proof-of-concept data are strong. Kim et al. designed a long-acting rifabutin depot using biodegradable polymers and biocompatible solvents that form a solid matrix after subcutaneous delivery. A single dose sustained therapeutic levels and prevented or treated TB in mice, greatly reducing dosing frequency [149]. Complementary overviews summarize development considerations, including excipients, depot formation, pharmacology, and device requirements [34].

Rifapentine is also progressing toward long-acting use. Preclinical nanoprecipitation spray-drying work shows encouraging depot characteristics and alignment with pharmacokinetic targets for long-acting rifamycins [150]. Surveys in high-burden regions report strong patient and provider support for less frequent dosing if safety is maintained, a factor that is important for real-world implementation [38].

Long-acting systems offer clear advantages but have limitations. Once administered, rapid reversal is difficult if toxicity occurs, pregnancy becomes a concern, or companion drugs are interrupted. A prolonged pharmacokinetic tail may encourage resistance if drug levels fall while bacteria persist [34]. This risk is particularly relevant when bacterial clearance is incomplete, as extended exposure to subtherapeutic concentrations can select for resistant subpopulations, a concern highlighted in analyses of long-acting antimicrobial therapies [151]. Controlled-release oral approaches, such as microencapsulated rifamycins, have produced sustained exposure and reduced toxicity in animals. These strategies keep open the possibility of once-weekly oral regimens that could complement injectable options [152].

### 4.5. Co-Delivery and Combination Platforms

TB therapy requires combinations, and nanoplatforms are evolving to match this need. Co-loaded systems deliver antibacterial drug and host-directed agents together and can time release for synergy. One example pairs rifampicin with autophagy-inducing compounds to improve intracellular killing and reduce harmful inflammation. Mannose-decorated inhalable micelles containing rifampicin and curcumin targeted alveolar macrophages and improved antibacterial activity in vitro [134]. Macrophage-targeted curcumin NPs alone induced autophagy and suppressed both drug-susceptible and drug-resistant strains [153].

Other groups have designed immunomodulatory NPs that trigger autophagy in infected macrophages and reduce lung bacterial burden in mice [154]. Additional work uses graphene-oxide systems to deliver rifampicin to macrophages, enhance intracellular killing, and shift macrophages toward an antibacterial M1 phenotype [100].

Recent reviews describe multi-payload carriers built from polymer, lipid, or hybrid materials. These carriers can deliver drugs sequentially or in combination and can incorporate imaging elements to track drug distribution [155]. Dual-payload systems show translational promise but complicate interpretation. Benefits may arise from targeting, release kinetics, or the host-directed component. Without factorial clinical trials or validated PD markers, superiority over optimized oral combinations remains unproven. Manufacturing complexity also increases as particle size, polydispersity, and cargo ratio must remain consistent through scale-up [28,132].

## 5. Bridging Therapeutics and Delivery: The Precision Pharmaceutic Paradigm

Progress in TB treatment now depends on combining potent agents with delivery systems that place drug exactly where it is needed. This approach defines precision pharmaceutics. It aligns drug potency with the time, place, and duration of exposure at the lesion. It designs delivery systems that match drug pharmacology and the biology of infection. It uses artificial intelligence (AI) and advanced modeling to co-optimize drug and delivery. It also plans for affordability, access, and adherence from the start of development. The guiding question is shifting from “What is the drug?” to “How will it be delivered, targeted, and completed by the patient?”

### 5.1. Case Studies: Improved PK/PD Through Delivery Innovation

Table 5 links each agent or formulation to its model, PK/PD outputs, and key implications. It shows how drug and delivery can be paired to improve lesion-focused performance. BDQ: Lyons et al. analyzed a 14-day early bactericidal activity trial to build a mechanistic PK/PD model of BDQ. With loading doses on days 1 and 2 followed by daily dosing between 100 mg and 400 mg, they estimated a maximum kill rate of 0.23 ± 0.03 log10 CFU/mL/day, an EC50 near 1.6 ± 0.3 mg/L, and an onset delay of about 40 ± 7 h. Simulations suggested that daily 200 mg, 300 mg, or 400 mg achieve about 40, 50, or 60 percent of maximal early bactericidal activity over 14 days. These findings highlight BDQ’s long half-life, large distribution volume, and the balance needed between sustained exposure and toxicity—features suited to innovative delivery [156].

Ajayi et al. addressed solubility and pediatric dosing by creating BDQ vegetable-oil nanoemulsions using design of experiments (DoE) within a quality-by-design framework. The optimized formulation produced droplets near 192 nm with a polydispersity index of about 0.12, a zeta potential near −26 mV, BDQ content near 3.1 mg/mL, and controlled release. Although preclinical, this work suggests better oral bioavailability and child-friendly formats [157]. Kaushik et al. developed an injectable BDQ depot. A single 160 mg/kg dose provided activity for 12 weeks in a validated preventive-therapy mouse model. One injection plus two weeks of oral BDQ, with or without high-dose rifapentine, matched or exceeded controls. These results show how depot delivery can flatten peaks, extend exposure, and simplify dosing [158].

Rifabutin: Kim et al. developed an in situ forming implant (ISFI) using PLGA and biocompatible solvents. Amphiphilic additives improved drug content and controlled polymer erosion. A single subcutaneous injection in mice sustained high plasma concentrations for about 16 weeks, prevented *M. tuberculosis* challenge, and cleared acute infection in the lung and extrapulmonary sites. This work reframes a backbone rifamycin as a near-quarterly option [149].

CFZ: Rongala et al. created an inhalable PLGA CFZ dry powder using spray-drying guided by DoE. The optimized powder (mean size near 1 µm; zeta potential near −31 mV) showed high entrapment, strong loading, acceptable aerosolization, and biphasic release. It also produced about eight-fold higher in vitro activity against *M. tuberculosis* H37Ra, consistent with a profile suited for alveolar macrophage targeting [133]. Stemkens et al. proposed a four-week, 300 mg once-daily oral loading approach to accelerate CFZ target levels, underscoring that dosing strategy is part of formulation science [159].

Liposomes and nanoparticles: Yildirim and Düzgünes reported that NP systems often produce tissue concentrations more than five-fold higher than free drug and improve survival in preclinical models evidence for macrophage homing in mycobacterial disease [160]. In guinea pigs, Garcia-Contreras et al. tested nebulized rifampicin liposomes and liposome–microparticle blends. Lungs showed significant CFU declines and improved spleen weights, supported by histology [161]. Khadka et al. showed that pulmonary delivery in mice and guinea pigs increases rifampicin bioavailability and lung exposure at lower doses than oral dosing [25].

Design for macrophage uptake: synthesizing work across many platforms, Kumar et al. outlined practical design cues. Carriers between about 100 nm and 200 nm with appropriate surface charge, stealthing, and pH-, redox-, or enzyme-responsiveness align closest with lesion-level PK/PD metrics more relevant to cure than plasma PK alone [28].

### 5.2. AI and Computational Modeling in Drug Delivery Co-Optimization

System performance depends on particle size and shape, surface charge, ligand density, release behavior, inhalation properties, body distribution, and lesion heterogeneity. Computational modeling in drug delivery serves two main purposes. First, it supports the design and optimization of new drug molecules using methods such as molecular docking, molecular dynamics, and structure activity or structure property relationships. Second, it supports the design and optimization of drug carriers and delivery systems. In this context, AI based tools are increasingly combined with classical molecular mechanics to guide polymer and excipient selection [162,163,164]. AI and machine learning (ML) now play a growing role in formulation decision making.

Jena et al. described models that predict formulation properties such as particle size and zeta potential, rank targeting ligands, and simulate polymer release, which reduces experimental workload [165]. As a practical example, active machine learning has been used to screen polymer composition and excipient ratios efficiently. This approach identified PLGA-PEG nanoparticle formulations with improved cellular uptake using only a limited number of experimental iterations [166]. Other reviews describe how AI is used to guide polymer selection, core shell design, and responsive carrier chemistry [167].

Modeling in TB addresses drug exposure at both organ and lesion levels, as well as airway deposition. Physiologically based pharmacokinetic (PBPK) frameworks estimate drug concentrations in tissues that are difficult to sample, including pleura and lymph nodes. These models help identify compartments where treatment response may be suboptimal [168]. Specific applications include BDQ exposure in central nervous system (CNS) TB and rifampicin dosing during pregnancy, where direct measurement is limited [169,170]. Lesion scale and imaging informed models explain drug trapping and diffusion barriers and show how physicochemical properties can be adjusted to improve penetration [9]. Agent based granuloma simulations further show how lesion diversity and PK variability influence the probability of bacterial clearance [171].

For inhaled tuberculosis therapy, computational fluid dynamics (CFDs) and particle simulations are used to predict interactions between the device, aerosol particles, and the airway. CT based modeling generates individualized deposition maps for dry powder inhalers and nebulized nanocarriers, which supports delivery to distal alveoli and macrophages [172]. Small changes in breathing pattern or particle size can shift deposition between airway regions, guiding formulation and device optimization [173]. Simplified PK tools are also emerging. The murine minimal PBPK model stormTB enables rapid evaluation of dosing scenarios for rifapentine, bedaquiline, and quabodepistat [174]. Related simplified PBPK approaches are under development to better integrate pharmacokinetics with formulation design [175].

A key limitation remains. Many predictions from AI, CFD, and PBPK models rely on plasma PKs or idealized deposition patterns rather than direct measurements from human lesions. Until prospective studies link predicted airway deposition and intralesional exposure with measured outcomes, such as imaging data or drug levels from bronchoalveolar lavage (BAL), these predictions should be interpreted with caution [9,12,176].

### 5.3. Strategies for Intracellular Penetration and Sustained Exposure

*M. tuberculosis* persists inside alveolar macrophages and within granulomas whose caseous, hypoxic, and acidic centers restrict diffusion. Plasma-adequate dosing can leave intralesional gaps. Newer formulations therefore emphasize macrophage targeting and sustained exposure [177]. Nanocarriers smaller than 200 nm that use ligands such as mannose, glucan, or hyaluronic acid raise intracellular drug levels and strengthen bactericidal activity while protecting labile compounds and reducing off-target effects [135]. Stimuli-responsive carriers match lesion conditions. pH-, redox-, or enzyme-responsive designs remain inert in circulating blood and release drug in acidic, oxidative, or enzyme-active niches within infected macrophages and caseum [176,178]. These profiles align with pH patterns seen in human and animal TB, where lung acidity increases during active disease and evolves with granuloma maturation [179].

Sustained exposure is the complementary strategy. TB grows slowly, and many niches are poorly perfused, so the aim is to maintain concentrations above minimum inhibitory or bactericidal thresholds without sharp peaks. Two illustrative anchors are a long-acting rifabutin ISFI that maintained therapeutic plasma concentrations for about 16 weeks after one injection and prevented or treated infection in mice [149], and a long-acting BDQ suspension that remained active for 12 weeks in a preventive-therapy mouse model [158].

Pulmonary sustained-release approaches bridge oral and parenteral depots. For rifampicin, aerosols and powders can increase lung concentrations at lower doses than oral regimens, raise macrophage uptake, and reduce systemic exposure. Human translation remains limited, but animal PK and PD and mechanistic rationale are strong [25].

### 5.4. Patient-Centered Issues: Adherence, Cost, Access

Precision pharmaceutics is useful only if patients can complete treatment. Attrition reflects long regimens, pill burden, and repeated clinic visits. Long-acting strategies reduce dosing frequency and visit demands. Surveys in high-burden regions report strong interest in less frequent dosing, particularly injectables, when safety, efficacy, and affordability are ensured [38].

Costs and logistics determine impact. High-technology systems will fail if they require specialized equipment, cold-chain transport, or costs beyond public-sector capacity. Designing for constraint from the outset heat-stable formulations, simple devices, and transport-stable products helps ensure that treatment can be completed. Preference studies show that with these factors addressed, acceptability of long-acting and inhaled options is high [38]. Aligning formulation with real-world context supports better completion and lower relapse [25,149].

### 5.5. Synthesis and Future Directions

Precision pharmaceutics integrates molecules, carriers, and delivery routes with lesion biology and real-world constraints. pH, redox, and hypoxia now inform projected site-of-action exposure before early clinical studies. Targeted nanocarriers, responsive platforms, long-acting depots, and inhaled powders can translate these projections into improved lesion exposure with fewer systemic effects [25,135,149,179]. The forward path is integration. PBPK and PD models set exposure goals. AI-guided formulation selects particle size, charge, ligands, and triggers to meet them. Iterative in vivo testing confirms exposure and tolerability. These steps make TB therapy more feasible to complete and support the long-term goal of shorter, safer, and more accessible regimens [25,38].

**Table 5:** Precision Pharmaceutics for TB: Verified case studies and design rules.

Agent/Theme	Approach	Model/Setting	Key Findings	Practical Takeaway	Refs.
BDQ: EBA PK/PD model	Mechanistic PK/PD re-analysis of 14-day EBA trial	Adults with drug-susceptible pulmonary TB; monotherapy dataset re-analyzed	BDQ produced measurable kill with estimated maximum rate near 0.23 log10 CFU/mL/day and EC50 near 1.6 mg/L	Exposure-aware dosing and delivery needed to balance efficacy and safety	[156,180]
BDQ: pediatric-leaning nanoemulsions	DoE-guided vegetable-oil nanoemulsion	Preclinical formulation work with in vitro release and stability	Droplets near 190 to 200 nm with controlled release and scalable processing	Supports child-friendly oral BDQ formats	[157]
BDQ: long-acting depot	Long-acting injectable suspension	Validated mouse preventive-therapy model	Single dose near 160 mg/kg protected for about 12 weeks	Depot delivery extends exposure and reduces dosing burden	[181]
Rifabutin: ISFI	PLGA-based in situ forming implant	Mouse prevention and treatment models	Sustained rifabutin for about 16 weeks and cleared infection	Quarterly style option for rifamycins	[149]
CFZ: inhalable powder	DoE-optimized PLGA microparticles	In vitro work with *M. tuberculosis* H37Ra and aerosol testing	Particles near 1 μm with high entrapment and biphasic release; eight-fold higher activity	Suited for alveolar macrophage targeting	[133]
CFZ: oral loading	Once-daily 300 mg for four weeks	Clinical PK setting	Faster attainment of CFZ target concentrations	Alternative when inhalation not feasible	[159]
Rifampicin: liposomes for the lung	Nebulized liposomes and microparticle blends	Guinea pig pulmonary TB model	Lower lung CFU and smaller spleen weights	Evidence for pulmonary translation	[161]
Rifampicin: inhaled powders	Aerosolized dry powders	Mouse and guinea pig PK/PD studies	Higher bioavailability and lung exposure at lower doses than oral dosing	Device and route can outperform oral dosing	[25]
Nanocarriers: design rules	Macrophage-targeted polymeric and lipid carriers with responsive release	Synthesis of nanocarrier evidence	Carriers between about 100 and 200 nm with tuned charge and contextual responsiveness raised intralesional levels	Lesion PK/PD predicts cure better than plasma; engineered carriers outperform free drug	[28]

## 6. Translational and Regulatory Challenges

### 6.1. Regulatory and Gmp Hurdles for Nanocarriers and Inhalables

Turning “drug + delivery” into usable products requires early quality and regulatory planning. In the United States, the FDA applies an evidence-based, product-specific approach for nanomaterial-containing medicines. Sponsors are expected to link critical quality attributes (CQAs), including particle-size distribution, surface chemistry, and kinetic stability, to clinical performance and to maintain these attributes across the product life cycle [142,182,183]. For liposomes, FDA guidance outlines expectations for CMC, PK and bioequivalence, and labeling, indicating that TB liposomes and similar nanosystems will face comparability requirements during development and scale-up [182].

For inhaled products such as metered-dose inhalers (MDIs) and DPIs, the FDA requires detailed characterization of aerodynamic particle-size distribution, emitted dose, device resistance, and in-use performance. In practice, TB aerosols and powders must be developed as integrated device formulation systems rather than as drugs alone [183]. In Europe, the European Medicines Agency (EMA) emphasizes similar principles for nanotechnology products, including strong characterization, comparability after process changes, and risk-based CMC approaches tailored to the platform [184]. The overarching message is clear: playbooks designed for traditional small molecules do not fit complex carriers or inhalables, and regulatory-ready CMC must begin early.

### 6.2. Toxicology, Stability, and Pharmacovigilance: Safety Beyond Plasma

Complex carriers raise biodistribution questions that standard toxicology packages may not capture. Reviewers look for local tolerability, including histopathology for depot systems, immune interactions in macrophage-targeted platforms, and the clearance patterns of carrier components, not only the active pharmaceutical ingredient (API) [142]. Stability must reflect real-world stressors: heat, humidity, and routine handling can shift particle size or aerosol performance and reduce exposure at the site of disease [183]. After launch, programs are expected to conduct active drug-safety monitoring and management (aDSM) so that adverse events linked to new drugs and regimens are recognized and resolved within routine care [185,186]. Regions with limited pharmacovigilance infrastructure may need targeted support to attribute and manage safety signals. Building that support into development prevents fragile or uneven rollouts.

### 6.3. Manufacturing Complexity and Cost: Designing for Constraint

The strengths of nanocarriers and inhaled systems—tight size control, staged assembly, and device coupling—also create manufacturing challenges. Scaling from gram-level work to industrial output can alter size distributions, entrapment efficiency, residual solvents, or shear-sensitive features. Each shift carries possible clinical implications if not controlled [132]. Two international tools now define a common language for scale-up. ICH Q13 provides guidance on continuous manufacturing, including validation and process control [187,188]. ICH Q12 outlines life-cycle change management and explains how established conditions and comparability protocols can maintain quality during post-approval changes [189,190]. Affordability determines whether a product can succeed in TB programs. Public tenders dominate procurement, and platforms that depend on rare excipients, specialized compressors, or cold-chain storage face cost and logistics barriers. Designing for standard equipment, feasible technology transfer, and stable room-temperature shelf life increases the chance of adoption [191].

There is still a gap between how some reports present “clinic-ready” products and what regulators require. Promising data often appear without control strategies aligned with ICH Q12 or Q13. When particle size, PDI, ligand density, or aerodynamic particle-size distribution are not tied to clinical performance and protected through comparability protocols, scale-up and site transfer risk uncontrolled shifts. The cost of goods is also rarely disclosed, leaving uncertainty about real-world feasibility for public TB programs [182,189].

### 6.4. Integration Within the WHO End TB Strategy Framework

The End TB Strategy aims for an 80 percent reduction in incidence, a 90 percent reduction in mortality, and the elimination of catastrophic costs by 2030 [192]. Products that reduce clinic visits, simplify monitoring, and align with national prequalification and logistics systems are most likely to scale [191]. Long-acting depots and simple inhaled approaches can lower patient and facility costs and support progress toward the “no catastrophic costs” target, provided price and supply remain practical for national programs [192].

### 6.5. Harmonizing Rules for Nanomedicines and HDTs

There is no single global standard for nanomedicine regulation. Agencies apply case-by-case science, often using cross-cutting guidance and requiring product-specific evidence for quality and safety. Analyses of the regulatory landscape highlight differences in definitions and data expectations that slow worldwide development [193]. The International Organization for Standardization (ISO) technical report ISO/TR 10993-22, while developed for medical devices, remains relevant. It advises developers to characterize nanomaterials, assess potential release, and evaluate toxicokinetics, recognizing that conventional assays may not fully apply to nanoscale materials [194]. HDTs require a different risk–benefit assessment. Immune modulation, chronic exposure, and combination use with antibiotics demand tailored safety evaluations and careful post-approval vigilance. Updated ICH Q2(R2) and ICH Q14 guidelines on analytical method life cycle and real-time release testing offer structured approaches for complex products that rely on modern, risk-based control strategies [195,196].

### 6.6. What a Workable Path Looks Like

A viable path begins with regulatory-ready CMC. Developers should map CQAs that drive performance particle size and PDI for nanocarriers and aerodynamic particle-size distribution, emitted dose, and device resistance for inhalables and embed these attributes into a quality-by-design (QbD) strategy tied to clinical targets [142,183]. Stability and usability must match programmatic settings. This includes testing under heat and humidity cycling, transport vibration, and straightforward user handling for sites with inconsistent power [183]. Development should pair with aDSM plans that include traceable device lots, simple reporting tools, and feedback loops so field signals trigger controlled changes under ICH Q12 rather than emergency repairs [189,197]. Affordability must remain central. Standard equipment, common excipients, and early regional technology transfer can help ensure pricing compatible with public-sector procurement [187].

**Figure 3:**
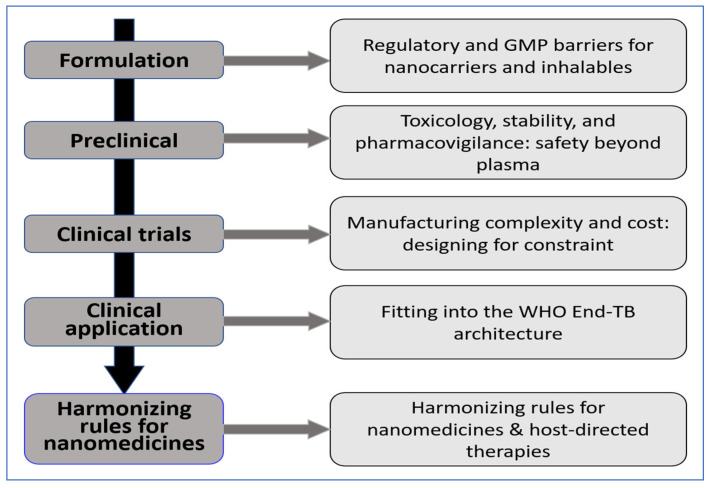
Translational pathway and implementation barriers for advanced TB drug delivery systems. The figure outlines the progression of advanced anti-TB drug delivery approaches from formulation design to clinical application. Early development must account for regulatory expectations and GMP requirements, particularly for nanocarriers and inhalable systems. Preclinical evaluation extends beyond plasma PKs to include toxicology, stability, and safety considerations relevant to pulmonary and intracellular exposure. During clinical development, manufacturability, cost, and scalability become key constraints. Successful implementation further requires alignment with the WHO End TB strategy and harmonized regulatory pathways for nanomedicines and host-directed therapies. These steps illustrate how scientific advances must be integrated with regulatory, manufacturing, and public-health considerations to enable real-world impact.

When sponsors, regulators, and TB programs align on these principles, delivery innovation moves faster and reaches the field more safely. These steps turn “drug + delivery” concepts into regimens people can complete [192]. Figure 3 illustrates the translational pathway from formulation development to clinical application, highlighting key challenges that influence whether advanced delivery systems can be implemented at scale. These include regulatory and GMP requirements, safety considerations beyond plasma exposure, manufacturability and cost constraints, alignment with the WHO End TB strategy, and the need for global regulatory harmonization.

## 7. Future Perspectives and Outlook

A practical goal is approaching: modeled, targeted, and finishable TB therapy that places the right drug at the right lesion for the right duration in a form people can complete. This future joins thoughtful formulation with patient-appropriate delivery, guided by rapid pathogen and host profiling and supported by manufacturing that public health systems can maintain. Many components are advancing; the priority now is to connect them so improvements appear in routine care rather than only in models [192].

### 7.1. Smarter Design and Selection

Data-driven methods can speed formulation decisions for complex carriers and inhaled systems. These approaches help match excipients, architectures, and processes to the CQAs that determine lesion-level exposure. They also simplify process development and analytics, improving “first-time-right” choices for DPIs, including aerodynamic distribution and emitted dose, and for nano-enabled systems, including size distribution and surface stability [198,199].

### 7.2. Delivery in Real Lungs

Patient-specific “digital-twin” airway models are becoming practical tools. These models combine computed tomography (CT) geometries with aerosol and particle dynamics and with physiology-based PK to forecast where an inhaled powder deposits, how much reaches distal airspaces, and how those patterns translate into time–concentration profiles at disease sites. In TB, where infection is patchy and intracellular, connecting smarter formulation with individualized deposition maps moves practice from exposure by assumption to exposure by design [200].

### 7.3. Choosing Regimens and Doses Sooner

The WHO now recommends targeted next-generation sequencing (tNGS) for rapid resistance detection, supported by a global interpretation portal. As laboratories expand whole-genome sequencing (WGS), direct comparisons are refining resistance-inference methods and cluster detection [201,202]. Host-guided dosing is also advancing. N-acetyltransferase 2 (NAT2)-guided isoniazid dosing can reduce hepatotoxicity and stabilize exposure. As point-of-care genotyping matures and additional pharmacogenes gain evidence, it becomes feasible to combine pathogen genotype, host genotype, and individualized inhaled dosing into a practical treatment plan [203].

### 7.4. Vaccines, Gene-Based Adjuncts, and Manufacturability

The first mRNA TB vaccine candidates, including BNT164, have entered Phase I trials. Efficacy remains unknown, but the platform allows faster iteration once safety and immunogenicity are established. Gene editing remains exploratory as therapy, but it already accelerates diagnostics and target discovery [39,204]. Sustained impact will require stable, transferable manufacturing. ICH Q13 supports continuous manufacturing, and ICH Q12 supports life-cycle change management. These tools help sponsors control cost, variability, and multi-site supply, which are essential for public-sector TB programs [187]. Sustainability and equity are central. Products must tolerate heat and transport, avoid cold chains when possible, fit procurement channels, and be straightforward to use. Otherwise, even effective candidates will not shift national outcomes [191,192].

### 7.5. A Near-Term Blueprint We Can Act on Now

A workable approach begins with modeling to narrow candidate formulations and identify the CQAs that determine lesion-site exposure. In first-in-human studies, this process should be paired with low-dose CT–based, patient-specific deposition maps to confirm that an inhaled dose reaches distal airways. Dosing and interval should follow PK/PD principles, and adjustments should proceed through comparability protocols under ICH Q12 rather than ad hoc changes. More precise regimen choices can reach the bedside through baseline tNGS or WGS and NAT2-guided isoniazid dosing, expanding to additional pharmacogenes as evidence grows. Manufacturing should follow ICH Q13 and ICH Q12 from the start so cost, process control, and multi-site supply stay aligned with clinical intent and national budgets [187,198,200,201,203].

Gaps and priorities remain. First, human trials must confirm that inhaled therapy improves culture conversion and relapse compared with optimized oral therapy. Preclinical results, including inhaled rifampicin, are strong, but human evidence is limited. Second, dual-payload nanocarriers require factorial testing to separate targeting effects from payload effects. Third, validated lesion-site biomarkers, including MALDI mass-spectrometry imaging and alveolar-cell PK, are needed to verify intralesional exposure. Fourth, for oxazolidinones and HDTs, patient-centered endpoints such as neuropathy-free survival should complement culture-based measures. Fifth, CMC should link directly to outcomes through ICH Q12 and ICH Q13 strategies, and cost-of-goods should be reported to assess feasibility for low- and middle-income countries [12,25].

**Figure 4:**
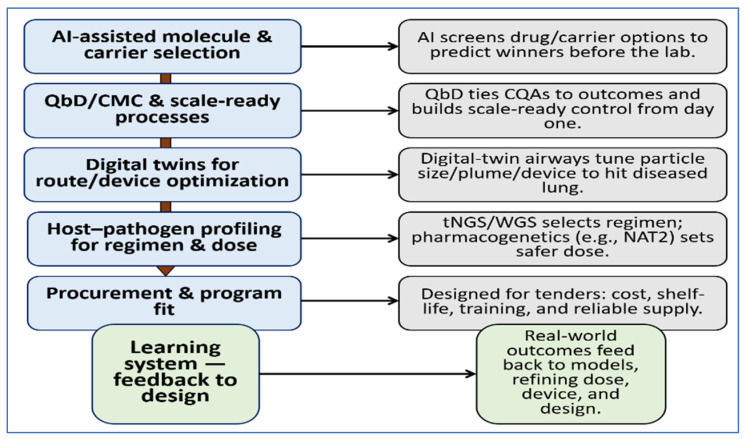
Practical roadmap and feedback loop for precision pharmaceutics in TB. This figure illustrates a stepwise framework linking drug and carrier selection, formulation design, delivery optimization, and clinical application. It highlights how administration strategies, formulation control through quality-by-design and CMC processes, and delivery optimization inform PK performance and programmatic feasibility. Importantly, the schematic shows a feedback loop in which real-world outcomes guide iterative refinement of formulation, dosing, and delivery approaches.

If these steps are combined, near-term progress could be substantial. Formulations can be chosen more rationally; inhaled options can be tuned to the lungs that need them; regimens can be selected using real resistance data at baseline; dosing can reflect each person’s genetic profile; and manufacturing can shift from central plants to regional lines without losing quality. The benefit is as much human as technical: fewer clinic visits, fewer missed wages, and regimens that work the first time. This is the promise of precision pharmaceutics in TB, innovation designed for the places and people who need it most [192]. Figure 4 outlines a practical roadmap for precision pharmaceutics in TB, highlighting how outcomes from real-world use feed back into refinement of formulation, dosing, and delivery strategies.

Beyond scientific performance, real-world success will depend on cost-effectiveness and the ability to manufacture at scale. Formulations that rely on complex processes, specialized excipients, or device-dependent delivery may be difficult to afford or produce widely if these issues are not addressed early. Designing products with scalable manufacturing, feasible regulatory pathways, and public-sector procurement in mind will be essential for meaningful impact in TB programs.

## 8. Conclusions

TB treatment is entering a pivotal phase. Standard multidrug regimens cure most drug-susceptible diseases but remain long, are often toxic, and do not reliably reach the microenvironments where *M. tuberculosis* persists. Evidence across this review supports a practical direction: pair potent therapeutics with delivery systems that improve intralesional and intracellular exposure. New small-molecule classes, including DprE1 and MmpL3 inhibitors, respiratory-chain blockers, and refined protein-synthesis inhibitors, expand options for both drug-susceptible and drug-resistant TB. Repurposed agents and next-generation oxazolidinones help balance efficacy with safety. Beyond traditional antibiotics, HDTs, antimicrobial peptides, monoclonal antibodies, phage strategies, and early CRISPR-based approaches offer complementary paths to enhance sterilization and limit tissue injury.

Delivery science links strong drugs to real-world cures. Inhalable powders and aerosols place drugs in the lung and increase uptake by alveolar macrophages with less systemic exposure. Nanocarriers protect fragile compounds, improve intracellular penetration, and release cargo in response to lesion cues, including low pH and redox gradients. Long-acting depots and implants can transform daily dosing into monthly or quarterly administration, reducing burdens for patients and health systems.

For these advances to matter, development should begin with the lesion in mind and remain anchored to what patients can complete. This includes the use of PK modeling, disciplined formulation, and early planning for manufacturability, affordability, and program fit. When potent therapeutics are aligned with targeted delivery and practical implementation, TB therapy can become shorter, safer, and genuinely finishable—helping more people heal and bringing communities closer to ending TB.

## Figures and Tables

**Table 1:** New-generation anti-TB drugs: mechanisms of action, and clinical status.

Drug	Drug Class	Primary Molecular Target/Mechanism of Action	Studied Population/Intended Role	Clinical Status	References
BDQ	Diarylquinoline	Inhibits mycobacterial ATP synthase (c-subunit), blocking ATP generation	MDR-/XDR-TB	Approved; WHO-recommended BPaL/BPaLM regimens	[1,49,50,51,52,53]
DLM	Nitroimidazole	F420-dependent prodrug inhibiting mycolic-acid synthesis after activation	DR-TB	Approved (EU/WHO)	[23,49,54,55,56]
Pa	Nitroimidazole	F420-dependent prodrug inhibiting cell-wall synthesis and generating reactive nitrogen species	MDR-/XDR-TB	Approved as part of BPaL/BPaLM	[24,56,57,58,59]
Quabodepistat (OPC-167832)	DprE1 inhibitor	Inhibits DprE1, blocking arabinan biosynthesis in the mycobacterial cell wall	DS- and DR-TB	Phase 2 (EBA/combination studies)	[45,60]
BTZ043	Benzothiazinone (DprE1 inhibitor)	Covalent inhibition of DprE1 leading to arabinan depletion	DS- and DR-TB	Phase 2	[56,61,62]
Telacebec (Q203)	Imidazopyridine amide	Inhibits cytochrome bc1 complex via QcrB	DS- and DR-TB	Phase 2a	[63,64]

**Table 2:** Developmental pipeline of anti-TB agents and molecular targets.

Candidate/Class	Primary Target/Mechanism of Action	Stage	Why It Matters	References
BDQ	ATP synthase (c-subunit) inhibition	Approved; BPaL/BPaLM anchor	Supports short oral DR-TB regimens; resistance through *Rv0678* and *atpE* requires DST and careful pairing	[58]
Pa	Nitroimidazole; F420-dependent activation with cell-wall effects	Approved in BPaL/BPaLM	Potent with BDQ and LZD; vulnerable to F420-pathway mutations	[118]
DLM	Nitroimidazole; F420-dependent activation	Approved (DR-TB)	Useful in shorter oral combinations; shares F420-pathway risks with Pa	[118]
Quabodepistat (OPC-167832)	DprE1 inhibition	Phase 2 (EBA; combinations)	First DprE1 candidate with human EBA and combination activity; DS-TB regimen under study	[45,60]
TBA-7371	DprE1 inhibition	Phase 2a EBA	Dose-dependent 14-day EBA; positioned for combinations	[61]
BTZ043	DprE1 inhibition (benzothiazinone)	Phase 2	On-target bactericidal activity; promising lesion penetration	[46,119]
Macozinone (PBTZ-169)	DprE1 inhibition	Early clinical	Covalent DprE1 inhibitor; ongoing PK/PD refinement	[120]
Q203	QcrB inhibition (cytochrome bc1 complex)	Phase 2a EBA	Human EBA; strong preclinical bactericidal activity; partner for energy-metabolism blockers	[63,64]
GSK3036656 (Ganfeborole)	LeuRS inhibition	Phase 2a EBA	First-in-class aminoacyl-tRNA synthetase inhibitor with human EBA	[47]
Sutezolid (PNU-100480)	Oxazolidinone; 50S protein synthesis	Phase 2b	Greater bactericidal effect than LZD; improved safety	[62,121]
TBI-223	Oxazolidinone; 50S protein synthesis	Phase 2	Retains efficacy with less neuropathy and myelosuppression; promising with BDQ and Pa	[79,122]
CFZ	Membrane and electron-transport effects	MDR/XDR-TB use	Improves outcomes; requires QTc monitoring and screening for Rv0678-linked cross-resistance with BDQ	[123]
LZD	Oxazolidinone; 50S protein synthesis	Approved (DR-TB)	Dose and duration adjustments preserve efficacy while reducing toxicity	[58,124]
Metformin (HDT)	AMPK and mitochondrial activation lead to autophagy	Cohort evidence; RCTs pending	Linked to lower mortality; strong macrophage-level support	[87,88]
Statins (HDT)	Enhance autophagy and phagolysosome maturation	Preclinical; mixed clinical findings	Reduce *M. tuberculosis* burden in models; translational potential	[90,125]
Vitamin D	LL-37 induction; immune modulation	RCTs/meta-analyses	Benefits mainly in deficient individuals; modest effects in others	[126]
LL-37 strategies	Direct antibacterial and immunomodulatory effects	Preclinical/translational	Nanodelivery improves stability and macrophage targeting	[102,127]
Anti-LpqH monoclonal antibody	mAb targeting LpqH on *M. tuberculosis*	Preclinical	Isotype-dependent protection; foundation for TB-focused ADC platforms	[105]
Mycobacteriophage DS6A	Phage-mediated lysis of *M. tuberculosis*	Preclinical	Active in macrophages and humanized mice; good manufacturing practice (GMP) development needed	[106]
CRISPR-TB (diagnostics)	Cas13/Cas12 detection of TB cell-free DNA	Research-grade assays	Enables rapid, non-invasive plasma cfDNA detection	[113,128]
LNP–mRNA (CysVac2)	mRNA platform inducing adaptive and innate responses	Preclinical; early human	Strong Th1 responses; candidate as BCG booster or standalone vaccine	[114]

**Table 3:** Composition and properties of inhalable particles for TB therapy.

Particle Composition	Typical Aerodynamic Size	Key Physicochemical Properties	Primary Biological Effect	Impact on Therapeutic Efficacy	References
Liposomes (phospholipid-based aerosols)	1–5 µm	High biocompatibility; encapsulation of hydrophobic drugs; sustained pulmonary residence	Uniform lung distribution and uptake by alveolar macrophages	Achieves bactericidal lung exposure at lower doses and reduces systemic toxicity in preclinical models	[25,127,128]
Solid LNPs/nanostructured lipid carriers	100–300 nm	Lipophilic core; physical stability; controlled drug release	Prolonged retention in lung tissue and intracellular compartments	Maintains local drug concentrations and supports intracellular killing	[27,28,128]
Polymeric NPs (e.g., PLGA)	100–300 nm	Tunable degradation; surface functionalization; ligand attachment (e.g., mannose)	Enhanced uptake by infected macrophages and granulomas	Improves intracellular delivery and lesion-level exposure compared with free drug	[28,128,132,133]
Dry-powder inhaler (DPI) microparticles	1–5 µm	Low moisture content; optimized dispersibility; formulation–device compatibility	Efficient deep-lung deposition without external power	Enables direct lung targeting and improves aerosol performance in translational formulations	[26,29,30]
Surface-modified particles (e.g., mannosylated systems)	100–300 nm	Ligand-mediated targeting; controlled surface charge	Preferential uptake by alveolar macrophages	Increases drug concentration at intracellular bacillary niches	[28,128,134]

**Table 4:** Stimuli-responsive delivery systems relevant to TB lesions.

System Type	Dominant Trigger in TB Lesions	Typical Design Feature	Primary Rationale
pH-responsive	Acidic pH in phagosomes and caseous necrosis	Acid-cleavable linkers or pH-sensitive polymer coatings (e.g., chitosan, PLGA blends)	Enables drug release in infected macrophages while remaining stable at physiological pH
Enzyme-responsive	Lesion-associated host or mycobacterial enzymes (e.g., esterases, proteases)	Enzyme-cleavable bonds within the carrier or linker	Improves selectivity by releasing drug preferentially in infected tissue
Redox-responsive	Elevated intracellular redox gradients and reactive oxygen species	Disulfide or thioketal bonds sensitive to redox conditions	Biases drug release toward intracellular compartments of infected cells
Multi-trigger systems	Combined acidic pH with redox or enzymatic cues	Layered or core–shell carriers integrating multiple responsive elements	Enhances spatial and temporal control of drug release

## Data Availability

No new data were created or analyzed in this study. Data sharing is not applicable.
